# Supplementary material for: Identification of photoperiod-induced specific miRNAs in the adrenal glands of Sunite sheep (Ovis aries)
Source: Front Vet Sci. 2022 Jul 22;9:888207. doi: 10.3389/fvets.2022.888207 (PMC9354845; doi:10.3389/fvets.2022.888207)
Supplement: Supplementary file 1 [file Data_Sheet_1.ZIP › Supplementary file/miRDeep2 file.zip!/miRDeep2 file/Know_Structure/SP42A3/oar-mir-136.pdf]

miRBase precursor : oar-mir-136
Total read count : 4973
oar-miR-136 read count : 1046
remaining reads : 3927

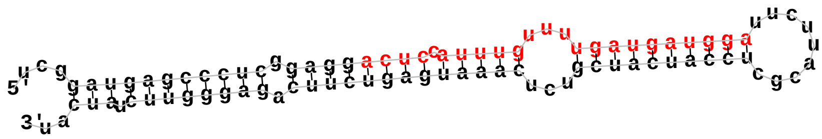

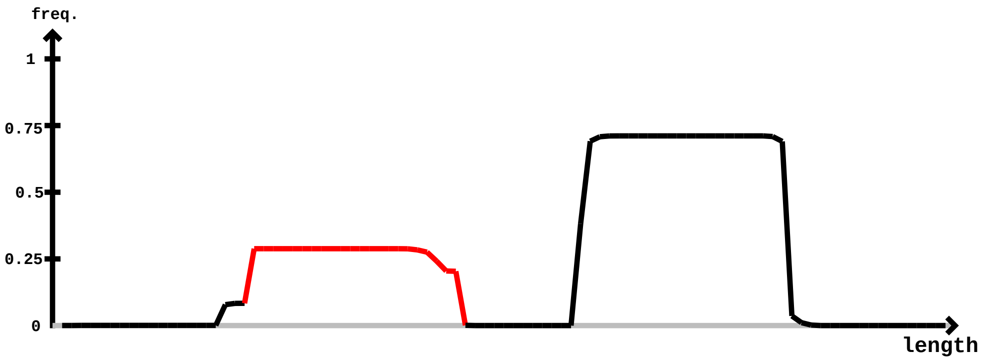

oar-miR-136

```
5'- ucggaugagcccucggaggacuccauuuguuuugaugauggauucuuacgcuccaucaucgucucaaaugagucuucagaggguucuaucau   -3'    exp
    ...(((((((((((.((((((((.(((((...((((((((((.........))))))))))...))))))))))))).)))))))).)))..    reads      mm         sample
    ucggaugagcccucgga...........................................................................    1          0          seq
    .cggaugagcccucggagg.........................................................................    2          0          seq
    ................aggacuccauuuguuuuga.........................................................    2          0          seq
    ................aggacuccauuuguuuugau........................................................    20         0          seq
    ................aggacuccauuuguuuugaug.......................................................    42         0          seq
    ................aggacuccauuuguuuugauga......................................................    162        0          seq
    ................aggacuccauuuguuuugaugau.....................................................    161        0          seq
    .................ggacuccauuuguuuugauga......................................................    5          0          seq
    .................ggacuccauuuguuuugaugau.....................................................    20         0          seq
    ..................gacuccauuuguuuugaugau.....................................................    1          0          seq
    ...................acuccauuuguuuugaugau.....................................................    2          0          seq
    ...................acuccauuuguuuugaugaug....................................................    6          0          seq
    ...................acuccauuuguuuugaugaugg...................................................    1007       0          seq
    ...................acuccauuuguuuugaugaugga..................................................    5          0          seq
    .....................................................caucaucgucucaaaugagu...................    11         0          seq
    .....................................................caucaucgucucaaaugaguc..................    72         0          seq
    .....................................................caucaucgucucaaaugagucu.................    1797       0          seq
    .....................................................caucaucgucucaaaugagucuu................    18         0          seq
    ......................................................aucaucgucucaaaugagu...................    1          0          seq
    ......................................................aucaucgucucaaaugaguc..................    19         0          seq
    ......................................................aucaucgucucaaaugagucu.................    1431       0          seq
    ......................................................aucaucgucucaaaugagucuu................    86         0          seq
    ......................................................aucaucgucucaaaugagucuuc...............    3          0          seq
    .......................................................ucaucgucucaaaugagucu.................    25         0          seq
    .......................................................ucaucgucucaaaugagucuu................    22         0          seq
    .......................................................ucaucgucucaaaugagucuuc...............    33         0          seq
    .......................................................ucaucgucucaaaugagucuuca..............    3          0          seq
    ........................................................caucgucucaaaugagucuuc...............    8          0          seq
    ........................................................caucgucucaaaugagucuuca..............    7          0          seq
    .........................................................aucgucucaaaugagucu.................    1          0          seq
```
